# Disinhibition-induced transitions between absence and tonic-clonic epileptic seizures

**DOI:** 10.1038/srep12618

**Published:** 2015-07-30

**Authors:** Denggui Fan, Qingyun Wang, Matjaž Perc

**Affiliations:** 1 Department of Dynamics and Control, Beihang University, Beijing 100191, China; 2 Department of Physics, Faculty of Science, King Abdulaziz University, Jeddah, Saudi Arabia; 3 Faculty of Natural Sciences and Mathematics, University of Maribor, Koroška cesta 160, SI-2000 Maribor, Slovenia; 4 CAMTP—Center for Applied Mathematics and Theoretical Physics, University of Maribor, Krekova 2, SI-2000 Maribor, Slovenia

## Abstract

Electrophysiological experiments have long revealed the existence of two-way transitions between absence and tonic-clonic epileptic seizures in the cerebral cortex. Based on a modified spatially-extended Taylor & Baier neural field model, we here propose a computational framework to mathematically describe the transition dynamics between these epileptic seizures. We first demonstrate the existence of various transition types that are induced by disinhibitory functions between two inhibitory variables in an isolated Taylor & Baier model. Moreover, we show that these disinhibition-induced transitions can lead to stable tonic-clonic oscillations as well as periodic spike with slow-wave discharges, which are the hallmark of absence seizures. We also observe fascinating dynamical states, such as periodic 2-spike with slow-wave discharges, tonic death, bursting oscillations, as well as saturated firing. Most importantly, we identify paths that represent physiologically plausible transitions between absence and tonic-clonic seizures in the modified spatially-extended Taylor & Baier model.

Absence and tonic-clonic seizures in epilepsy are two types of primary generalized seizures^1^,^2^,^3^,^4^, which represent the pathological brain rhythms involving the whole cortical region in humans. They can be typically distinguished by their inherent features. For grand-mal seizures, there exists a tonic phase in the electroencephalograph (EEG)^3^, which can be characterized by the neural paroxysmal fast activity with high frequency (>13 Hz) and low amplitude. And, clinical manifestation has shown that it can be characterized by the tense of muscles^5^, typically preictal awareness and consciousness^6^, and invariably postictal unconsciousness^7^. Furthermore, the EEG also shows that tonic oscillation can be generally followed by the evolution into low frequency and high amplitude slow-wave oscillation, which is also known as clonic phase of epileptic seizures^2^,^3^. Studies have shown that absence epilepsy that mainly occurs in children and adolescents, is a petit-mal seizures, whose EEG and clinical features are evidently different from those of tonic-clonic seizures^8^,^9^,^10^,^11^,^12^,^13^,^14^. In the absence seizures, patients’ consciousness can be suddenly and transiently deprived^6^ along with the EEG characteristics of periodic spike and slow-wave (~2–4 Hz) complexes^15^. Compared to humans, rodent models for the absence seizures in epilepsy can generate spike and slow-wave oscillations in the relatively fast frequency (5–10 Hz)^16^, which has been demonstrated to be dependent on the GABAergic conductances of the relay neurons in thalamus^16^,^17^,^18^,^19^,^20^,^21^. Generally, periodic spike and slow-wave discharges are bilaterally synchronous through clinical observations, and also well structured^2^,^8^.

It is generally believed that epileptic seizures including tonic-clonic and absence seizures can be induced by the abnormal interactions within the corticothalamic system composed of the cerebral cortex and thalamus^18^,^20^,^22^,^23^,^24^,^25^,^26^,^27^,^28^,^29^,^30^,^31^. This can be supported by the simultaneous recordings from the cortex and thalamus of either the rodent animal models^32^ or clinical patients^33^. Due to the common anatomical nature, both the tonic-clonic and absence seizures can usually alternatively appear in one complete recording from the same patient^15^,^34^,^35^. The evidences from clinical experiments have demonstrated that tonic-clonic activity can evolve from absence seizure^36^, and a tonic seizure that can be accompanied by the absence seizure^37^. Hence, absence tonic-clonic seizure and tonic absence seizure have been termed as the two typical type of seizures^36^,^37^.

We know that firing behaviors of neural systems are very complex due to the complexity of every neuron and the influence from the surrounding nerve cells. Investigations of the computational model can uncover some mechanisms of neural behaviors and their transitions including absence and tonic-clonic seizures^38^. Some literatures related to the neuronal network have focused on the research of brain activity, the magnitude of neurons and simplified sparse connections within the local brain dynamics^39^,^40^,^41^. However, it is still intractable to develop good computational model, which can be matchable to the clinical and experimental results of realistic brain dynamics. As a low computation and complexity alternative, the neural field model can be used to describe the large-scale dynamics of neuronal populations within the cerebral cortex that reproduces the realistic firing rates for each neuronal population^1^,^42^. Because it is over-simplified, the neural field model can not yet represent realistic evolutions of the neural population activities and its spatial distribution.

It is well known that the spatially-extended neural field model network can be taken as a compromise^43^,^44^,^45^,^46^. It is composed of neural field model representing the large-scale dynamics^47^ of local cerebral cortex on various distantly and globally distributed space locations. Due to its validity on both the local and global scales, the spatially-extended field model network can well produce typical dynamical behaviors of neural systems, which can be a good prototype of macroscopic absence seizure in epilepsy^43^. Both the absence and tonic-clonic seizures are pathological nonlinear phenomena in the EEG of patients with epileptic disorders. Up to now, their spatiotemporal features have not yet been well understood. Presently, we develop computational mathematical models to provide the way to investigate macroscopically the hypersynchronized pathological behavior of epilepsy seizures. In particular, we study the transition dynamics of absence and tonic-clonic seizures, and the spatiotemporally synchronous evolutions of epileptic seizures by means of an isolated modified Taylor & Baier model, and the spatially-extended modified Taylor & Baier model.

## Results

**Figure 1:**
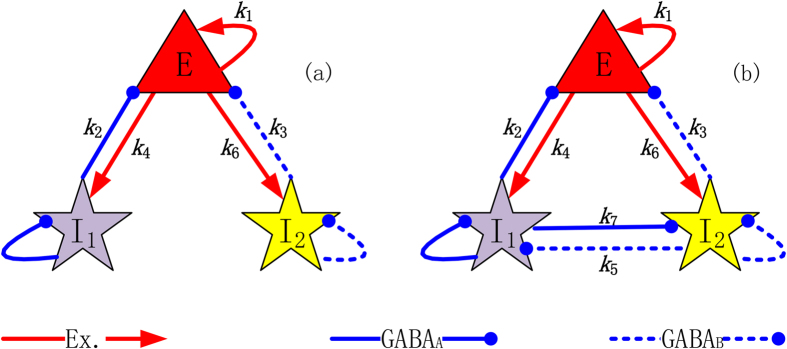
Schematic plots of the studied models. (**a**) the Taylor & Baier model. (**b**) the modified Taylor & Baier model constructed by three neuronal populations with one excitatory population E and two dependent inhibitory ones, I_1_, I_2_. Here, the I_1_ and I_2_ are mediated by the fast and slow time scales of the inhibition receptors GABA_*A*_ and GABA_*B*_, respectively. Excitatory connections are shown in red solid arrows. Inhibitory connections are shown in blue lines with closed ellipses, where solid and dashed ones represent the synaptic functions mediated by the GABA_*A*_ and GABA_*B*_, respectively.

**Figure 2:**
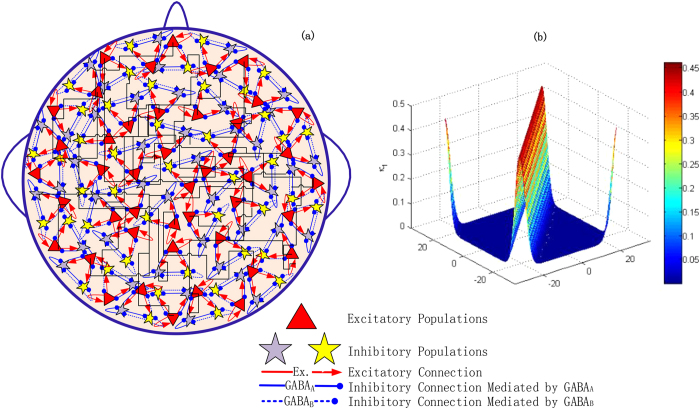
The spatially-extended Taylor & Baier network. (**a**) The network is composed of several single modified Taylor & Baier models, which is analogous to the procedure of real electroencephalograph (EEG) measurement. (**b**) The internal connection within the spatially-extended Taylor & Baier network adopted in the mode of “Mexican hat” connectivity. Without loss of generality, only the simulation of connectivity function *κ*_1_ in model network (Eq. (4)) is shown, which is constructed by using the periodic boundary conditions with an interval of 0.5, within [−25, 25]. This is equivalent to the number of spatial locations considered in this text.

We first modify the original model of Taylor & Baier^43^ (see Fig. 1(a)) by introducing the inhibition effects between two inhibitory populations I_1_ and I_2_ (see Fig. 1(b)). And then, based on electrophysiological experiments, we extend a spatial Taylor & Baier network model (see Fig. 2) to investigate its transition dynamics, which can be related to the epileptic seizures. Importantly, the transitional dynamics between absence and tonic-clonic seizures will be indeed explored. In particular, we will consider the mutually disinhibitory functions between I_1_ and I_2_, i.e., parameters *κ*_5_ and *κ*_7_, to study the transitional dynamics of the single modified Taylor & Baier model and spatially-extended network, respectively, as the disinhibitory effect is introduced.

### Transition dynamics in an isolated modified Taylor & Baier model

**Figure 3:**
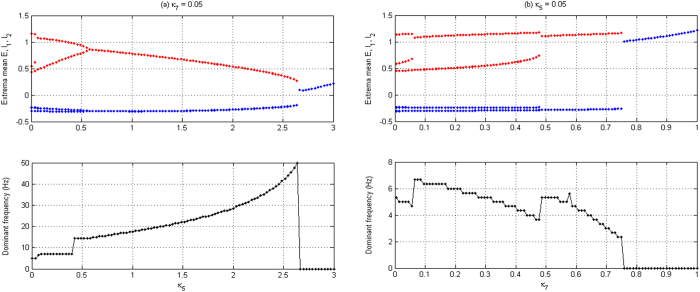
The bifurcation diagrams and their corresponding dominant frequencies for the single modified Taylor & Baier field model as the coupling strength (**a**) *κ*_5_ varies with *κ*_7_ = 0.05, and (**b**) *κ*_7_ varies with *κ*_5_ = 0.05, respectively. It can be found that the periodic 2-spike with slow-wave discharges, the 1-spike with slow-wave discharges, the simple tonic discharges, tonic death oscillations and statured firing can appear as *κ*_5_ and *κ*_7_ change.

In this section, we mainly investigate the dynamics of the isolated modified Taylor & Baier model induced by the mutually disinhibitory functions as the parameters *κ*_5_ and *κ*_7_ change. Results of Fig. 3 show that the single modified Taylor & Baier model can display rich dynamics as the inhibitory coupling strength *κ*_5_ or *κ*_7_ varies. In particular, from the upper panel of Fig. 3(a), it can be clearly seen that the dynamics of the modified Taylor & Baier model firstly transits from the periodic 2-spike and slow-wave discharges to the 1-spike and slow-wave discharges as the parameter *κ*_5_ changes with fixing *κ*_7 _= 0.05. Afterwards, transition to the simple tonic discharges can be found with further increasing *κ*_5_. For the larger value of *κ*_5_, the single modified model can exhibit tonic death oscillations, which occurs around *κ*_5_ = 3. Similarly, when we take *κ*_5_ = 0.05, bifurcation scenario of an isolated modified Taylor & Baier model is plotted in Fig. 3(b) as the parameter *κ*_7_ varies. Obviously, we can also find rich transitions from the periodic 2-spike with slow-wave discharges to the 1-spike with slow-wave discharges, finally to saturated firing as the parameter *κ*_7_ increases. In addition, variations of the corresponding dominant frequency are also given in the lower panels of Fig. 3, where we can observe two sudden jumps and one sudden dive in frequencies corresponding to the transitions of different firing states. Moreover, compared Fig. 3(a) with Fig. 3(b), it can be found that as *κ*_7_ is fixed, the inhibitory effect of I_2_ on I_1_, i.e., *κ*_5_ can induce high-frequency oscillations, while the inhibitory action of I_1_ on I_2_, i.e., *κ*_7_ can lead to low-frequency oscillations with step-likely decreasing frequencies when *κ*_5_ is fixed. Hence, both *κ*_5_ and *κ*_7_ can induce evident and stable periodic spike and slow-wave discharges, which are the key EEG feature of the pathological absence seizure in epilepsy. Also, moderate values of *κ*_5_ and *κ*_7_ can produce simple tonic (see Fig. 3(a)) and slow-wave (clonic) oscillations (see Fig. 3(b)), respectively.

**Figure 4:**
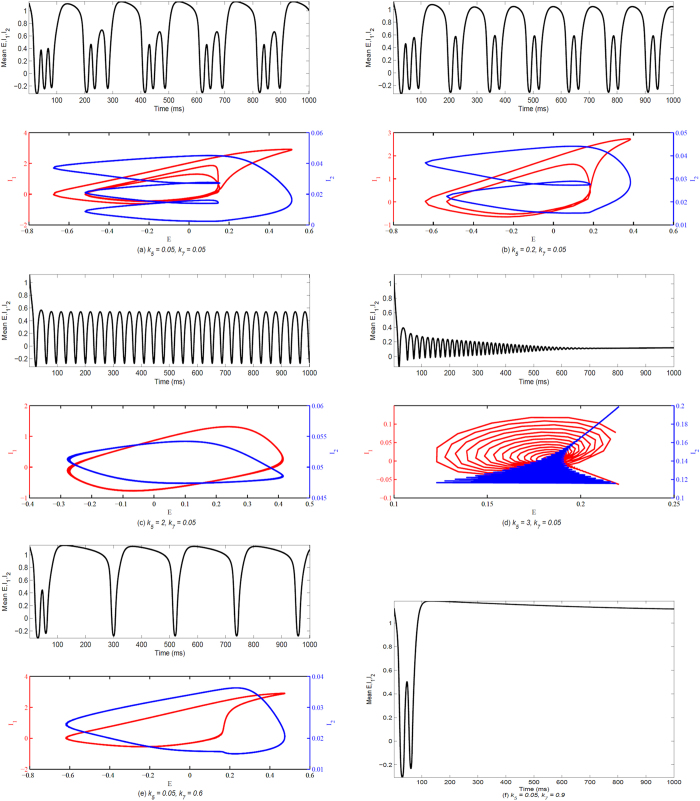
(Upper panels) The time series of the spatial evolution of all the excitatory populations E and the two inhibitory populations I_1_, I_2_; (Lower panels) The phase-plane portraits for the excitatory neuronal populations E verse the inhibitory neuronal populations I_1_ (red) and I_2_ (blue). The corresponding parameters are set as: (**a**) *κ*_5_ = 0.05, *κ*_7_ = 0.05 (the periodic 2-spike with slow-wave discharges), (**b**) *κ*_5_ = 0.2, *κ*_7_ = 0.05 (the periodic 1-spike with slow-wave discharges), (**c**) *κ*_5_ = 2, *κ*_7_ = 0.05 (the simple tonic discharges), (**d**) *κ*_5_ = 3, *κ*_7_ = 0.05 (tonic death oscillations), (**e**) *κ*_5_ = 0.05, *κ*_7_ = 0.6 (high-amplitude low-frequency slow-wave (clonic) oscillation). (**f**) The temporal evolution of the spatial mean of all the excitatory populations E and the two inhibitory populations I_1_, I_2_, with *κ*_5_ = 0.05, *κ*_7_ = 0.9 (saturated firing).

For a clearer vision, Fig. 4 shows the typical time series of the spatial mean of the excitatory populations E and the two inhibitory populations I_1_, I_2_, and corresponding phase-plane portraits of E verse I_1_ and I_2_, respectively with some combinations of the parameters *κ*_5_ and *κ*_7_. Particularly, as *κ*_7_ = 0.05, we take *κ*_5_ = 0.05, 0.2, 2, 3, respectively. And then, we can observe the complex transitions from the periodic 2-spike with slow-wave discharges (Fig. 4(a)), periodic 1-spike with slow-wave discharges (Fig. 4(b)) to the low-amplitude high-frequency tonic discharge (Fig. 4(c)), and finally to the tonic death oscillation (Fig. 4(d)), respectively. Also, as we fix *κ*_5_ = 0.05, apart from the dynamics similar to the above, we can observe other discharge patterns such as high-amplitude low-frequency slow-wave (clonic) oscillation (Fig. 4(e)) and saturated firing (Fig. 4(f)) as *κ*_7_ = 0.6 and 0.9, respectively. Hence, it is concluded that increasing *κ*_5_ can initially induce the transition from periodic spike with slow-wave discharges representing absence seizure to tonic epileptic seizure, and finally to the termination of epileptic seizures characterized by the tonic death oscillation. And, the larger *κ*_7_ can eventually lead to the saturated discharge of neuronal populations.

**Figure 5:**
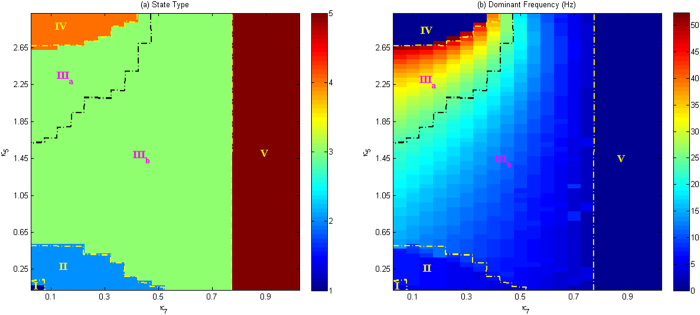
Different firing states (**a**) and variations of corresponding dominant frequency (**b**) of the single modified Taylor & Baier model are shown on the parameter plane (*κ*_5_, *κ*_7_), where we can observe rich firing states such as periodic 2-spike slow-wave discharges denoted by I, periodic 1-spike slow-wave discharges (II), tonic firing (III_a_), slow-wave (clonic) oscillations (III_b_), tonic death (IV) and saturated firing (V).

In order to investigate the overall dynamics transition of an isolated modified Taylor & Baier model as shown in Fig. 5, we depict the state transitions (Fig. 5(a)) and corresponding dominant frequency (Fig. 5(b)) of this isolated modified Taylor & Baier model as the two inhibitory coupling strengths *κ*_5_ and *κ*_7_ are changed. In particular, it can be seen in Fig. 5(a) that there are five types of firing states, which are denoted as: I: periodic 2-spike with slow-wave discharges, II: periodic 1-spike with slow-wave discharges, III: simple oscillations, IV: tonic death oscillation and V: saturated firing. Furthermore, for the type III: simple oscillations, according to the oscillatory frequency (Fig. 5(b)), it can be classified into III_a_: low-amplitude (~ ≤ 0.6, similarly hereinafter) and high-frequency (~ ≥ 25 Hz, similarly hereinafter) tonic, and III_b_: high-amplitude and low-frequency slow-wave (clonic) oscillations. For small *κ*_5_ and *κ*_7_, the system exhibits the 2-spike slow-wave discharges in the state ‘I’ as shown in the left bottom of Fig. 5(a). And, as they become larger, the system firstly transits to the ‘II’ state, and successively to the ‘III_b_’ with the slow-wave (clonic) discharge. With the further increasing *κ*_5_ and *κ*_7_, the system can transit either to the state ‘V’, saturated firing for all the *κ*_5_ and when *κ*_7_ is above 0.75, or to the states ‘III_a_’ and ‘IV’ as the *κ*_7_ < 0.5 and *κ*_5_ increases successively through the boundaries of these two states, respectively. Still, when the *κ*_5_ and *κ*_7_ lie in the state ‘III_a_’ where whether decreasing *κ*_5_ or increasing *κ*_7_, i.e., weakening the disinhibitory coupling action of I_2_ on I_1_ or strengthening the disinhibitory function of I_1_ on I_2_, can induce the state ‘III_b_’. According to the electrophysiological EEG observation, this can imply the transition from tonic to clonic seizures in epilepsy. Hence, state ‘III’ consisting of the ‘III_a_’ and ‘III_b_’ represents the parameter region of tonic-clonic seizure, which is a specific type of epilepsy seizure^1^,^48^. Also, the transition from state ‘II’ that indicates the physiologically epileptic absence seizure, to the state ‘III’ with the tonic-clonic seizure, or vice versa, is consistent with the electrophysiological observation for the patients with pathological epileptic behavior^36^,^37^. Moreover, for the small *κ*_7_, the *κ*_5_ can eventually induce the transition to the state ‘IV’: tonic death, which shows the seizure termination of pathological epileptic behavior described by this isolated modified Taylor & Baier model. But the larger *κ*_7_ can eventually lead to the saturated firing irrespectively of the value of *κ*_5_.

### Firing transitions in the spatially-extended modified Taylor & Baier model

Based on the Taylor & Baier model^43^ and the Amari model^49^, we use the mode of “Mexican hat” connectivity (see Fig. 2(b)) to spatially expand the modified Taylor & Baier neural field network model on several space locations. The spatially-extended Taylor & Baier network model is illustrated as in Fig. 2(a). This spatially-extended network model can match the process of electrophysiological EEG measurement, where every individual Taylor & Baier model on the specific location is analogous to the cortical contact of every electrode used in the EEG measurement. This idea results from the fact that an isolated Taylor & Baier model is a neural field model that represents the activity of neuronal populations, which is equivalent to the space scale of local cerebral cortex of the electrode contacts. More importantly, this network dynamics model can provide a novel way to reveal the firing characters of neuronal populations on different locations, and macroscopically investigate the spatiotemporal behaviors such as pathological synchronization of the cerebral cortex.

**Figure 6:**
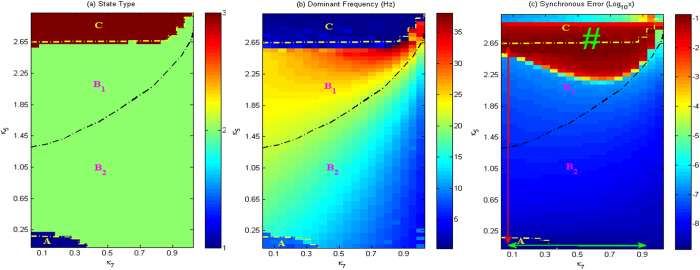
Different firing states (**a**) variations of corresponding dominant frequency (**b**) and the synchronous error (se) (log_10_se) (**c**) are respectively exhibited for all the neuronal populations E, I_1_, I_2_ and excitatory neuronal populations E of the spatially-extended Taylor & Baier model on the parameter plane (*κ*_5_, *κ*_7_). In particular, A denotes periodic 1-spike slow-wave discharge, B_1_ is tonic, B_2_ is slow-wave and bursting oscillation is indicated by C as *κ*_5_ and *κ*_7_ are changed in considered regions of the present paper. The red vertical and green horizontal arrows represent the possible transitional paths between absence seizure and tonic-clonic seizures.

Next, we will explore firing dynamics of the spatially-extended network as some key parameters are changed. As depicted in Fig. 6(a), we can find three types of firing states, which are indicated as, A: 1-periodic spike with slow-wave discharges, B: simple oscillations and C: periodic bursting oscillations as the parameters *κ*_5_ and *κ*_7_ vary in the parameter region considered. Further investigations show that the region of ‘B’ can also be divided into two subregions according to the corresponding dominant frequency (see Fig. 6(b)), i.e., B_1_: low-amplitude and high-frequency tonic discharge and B_2_: high-amplitude and low-frequency slow-wave (clonic) oscillation. It is important to explore the physiological significance of transitions of various dynamical behaviors. Similar to the dynamical transitions of an isolated Taylor & Baier model, the transition from B_1_ to B_2_ looks like to be the shift of tonic-clonic, but actually can be not a physiological transition. It is because the fact that a real shift of tonic-clonic seizure in epilepsy is generally accompanied by the transition of asynchronous activity to coherent oscillations, even if this transitional mechanism remains yet unknown. However, Fig. 6(c) gives the synchronous evolution induced by *κ*_5_ and *κ*_7_, which is expressed by the log_10_x-transformed synchronous error (LTSE) for all the excitatory neuronal populations E. For most of the parameter regions, the network system shows super-synchronous state, in where the LTSE is lower than −6. But, for the region signed by ‘#’, LTSE is around −2, which represents relatively weak synchronous or asynchronous state^50^. From Figs. 6(a–c), it can be inferred that overlapping island belonging to both the region of B_1_ and the #-signed one, represents a weak synchronous tonic discharge. In addition, absence seizure can be characterized by super-synchronization and well structured periodic spike with slow-wave discharges. Hence, the paths indicated by the red or green double-headed arrows, as shown in Fig. 6(c), are possible biological transition paths from tonic-clonic to absence seizures (descendant or left-facing paths) and vice versa (ascendant or right-facing paths), because it is accompanied by the transitions from asynchronous activity, coherent oscillations and to super-synchronous state, respectively. Actually, some kinds of transitional paths can also be figured out within the parameter plane of (*κ*_5_, *κ*_7_), which depends on the specific physiological environment.

**Figure 7:**
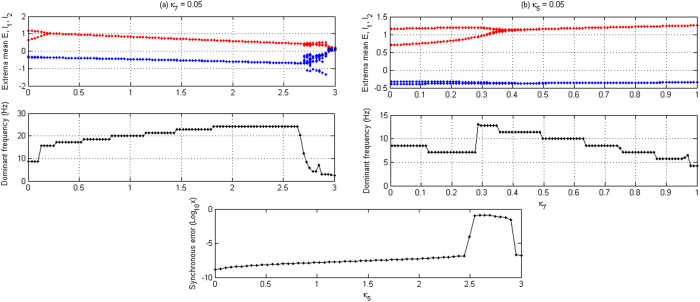
The bifurcation diagrams of firing states (**a**,**b**: Upper panels) and the evolutions of their corresponding dominant frequencies (**a**,**b**: Middle panels) and synchronous errors (**a**: Lower panel) are illustrated for the spatially-extended Taylor & Baier model as the coupling strength (**a**) *κ*_5_ changes with *κ*_7_ = 0.05, and (**b**) *κ*_7_ varies with *κ*_5_ = 0.05. It can be found that the periodic spike with slow-wave discharges, simple oscillations and periodic bursting oscillation can occur as *κ*_5_ and *κ*_7_ change.

To better understand the firing transition, we plot typical bifurcation diagrams to explore this along the two paths indicated by the double-headed arrows (see Fig. 6(c)) with fixing *κ*_5_ = 0.05 and *κ*_7_ = 0.05, respectively. Fig. 7 shows the bifurcation diagrams (Upper panels) of different firing states, accompanied by the evolutions of their corresponding dominant frequencies (Middle panels) and synchronous errors (Lower Panels, see Fig. 7(a)), as the parameters *κ*_5_ and *κ*_7_ are independently changed, respectively. Compared Fig. 7 with Fig. 3, we can find that 2-spike slow-wave discharges and saturated firing disappear due to the interactions of neural populations within the Taylor & Baier network with only stable 1-periodic spike with slow-wave discharges and tonic discharge being kept. Meanwhile, the parameter intervals of periodic spike with slow-wave discharges are decreased while the ones of tonic discharge are increased. In addition, the dominant frequencies are reduced with *κ*_5_ increasing but become high as *κ*_7_ increases, as compared Fig. 7 with Fig. 3. Specially, it is noted that the spatially-extended neural field model network is of having the spatio-temporal pattern. As shown in the lower panel of Fig. 7(a), we can observe the changes of synchronous errors for the model network as *κ*_5_ increases. For the ascendant case of vertical path (and vice versa) in the Fig. 6(c), corresponding to the lower panel of Fig. 7(a), the log_10_*x*-transformed synchronous error (LTSE) gradually climbs up with *κ*_5_ increasing. However, around *κ*_5_ = 2.5, there exists a sharp jump entering into the “plateau phenomenon” of LTSE corresponding to the transient asynchronous state. Along with this, compared to the upper and middle panels of the Fig. 7(a), the firing state of the model network transits from the initial super-synchronous periodic spike with slow-wave discharges to the asynchronous level of simple tonic oscillation. Finally, there exists a sharp dive in the synchronous error which corresponds to the synchronous bursting activity. Similarly, for the right-facing case of horizontal path in the Fig. 6(c), we can see the transition from periodic spike with slow-wave discharges to the simple slow-wave (clonic) oscillation (see the upper and middle panels of the Fig. 7(b)) of the spatially-extended model network with *κ*_5_ increasing.

**Figure 8:**
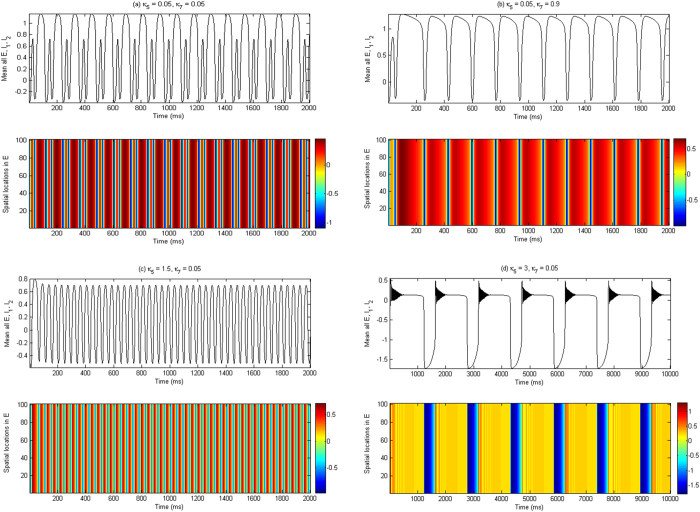
(Upper panels): The time series of the spatial mean for all the excitatory populations E and the two inhibitory populations I_1_, I_2_; (Lower panels): The spatiotemporal distribution for all the excitatory neuronal populations E, which corresponds to the Fig. 6 is shown as (**a**) *κ*_5_ = 0.05, *κ*_7_ = 0.05 (a periodic spike with slow-wave discharges), (**b**) *κ*_5_ = 0.05, *κ*_7_ = 0.9 (high-amplitude low-frequency slow-wave (clonic) oscillation), (**c**) *κ*_5_ = 1.5, *κ*_7_ = 0.05 (low-amplitude high-frequency tonic oscillation), (**d**) *κ*_5_ = 3, *κ*_7_ = 0.05 (periodic bursting oscillation), respectively.

As typical examples, we choose several parameters on the two transition paths (see Fig. 6(c)) to visualize the firing behaviors in different types of state regions, as shown in Fig. 8, where Figs. 8(a,c,d) correspond to the vertical path while Figs. 8(a,b) correspond to the horizontal path. As *κ*_5_ = 0.05, *κ*_7_ = 0.05, which corresponding to the region ‘A’, time evolution of the network clearly displays the periodic spike and slow-wave discharges (see Fig. 8(a)), Figs. 8(b,c) show the slow-wave (clonic) oscillation and tonic discharge, which correspond to the regions ‘B_2_’ and ‘B_1_’ with (*κ*_5_, *κ*_7_) = (0.05, 0.9) and (1.5, 0.05), respectively. Interestingly, as (*κ*_5_, *κ*_7_) = (3, 0.05) within the region ‘C’, the network can behave as the periodic bursting oscillations (see Fig. 8(d)). This can be caused by the over-strong inhibition of I_2_ on I_1_, i.e., large *κ*_5_ = 3. In addition, from the lower panels of Fig. 8, we also see the highest level of activities for the excitatory neuronal populations E during the periodic spike and slow-wave discharges, while the tonic, slow-wave (clonic) and periodic bursting oscillations show the relatively low level of activities of excitatory populations.

Notedly, even though the Taylor & Baier model or its spatially-extended network can display various dynamical behaviors, it is our interest to physiologically represent the electrophysiologically observed transitions between tonic-clonic and absence seizures in epilepsy. By means of the above investigation of disinhibitory effect within the modified Taylor & Baier model, we can indeed explore the disinhibition-induced synchronous evolution and the physiological transitions between tonic-clonic and absence seizures in epilepsy.

## Discussion

Based on the proposed dynamic models of neural populations, we have investigated the disinhibitory effect of inhibitory variables I_1_ and I_2_ on firing transitions of neural populations. The results have revealed that an isolated Taylor & Baier neural field model can show rich dynamical transitions including periodic spike with slow-wave discharges, simple tonic and slow-wave (clonic) oscillations as disinhibitory coupling strengths between I_1_ and I_2_ are changed. In addition, we further studied dynamics transition of the spatially-extended Taylor & Baier network consisting of several units in the “Mexican hat” connectivity as used in the original Taylor & Baier model or the original Amari model. Due to the interactions within the Taylor & Baier network, it is shown that the network can only show the transition of stable periodic spike with slow-wave discharges and tonic and clonic slow-wave oscillations as compared to the case of a single modified Taylor & Baier model. Importantly, both the single Taylor & Baier model and the spatially-extended network can produce stable periodic spike with slow-wave discharges, tonic and clonic slow-wave oscillations on their respective specific parameter regions, which characterize physiological transitions between absence and tonic-clonic seizures of epileptic disorders.

In addition, in the region signed by ‘#’ in Fig. 6(c), which corresponds to the moderate disinhibition effect of I_1_ on I_2_ and strong disinhibition function of I_2_ on I_1_, the modified Taylor & Baier network can exhibit the asynchronous or weak synchronous behaviors. For some other regions, the network has high level of synchronization or super-synchronous state. Because electrophysiologically observed tonic-clonic shift is accompanied by evolution from asynchronous activity to consistent oscillation, and absence seizure is characterized by super-synchronous periodic spike and slow-wave discharges, our results about the transitional routes between absence and tonic-clonic seizures in epilepsy can give some theoretical insights into understanding these two primary generalized epileptic seizures.

However, the mechanisms under the electrophysiologically observed transitions between generalized absence and tonic-clonic seizures are very complex and should be further explored in future. Such a complex nonlinear behavior of absence-tonic clonic(TC) transition can’t be caused by only the cerebral cortex but also the subcortical structures, e.g., absence seizure are known to arise in thalamo-cortical networks^25^,^26^,^27^,^28^,^29^,^30^,^31^ and basal ganglia plays a key regulation function in the absence seizure^42^ hence possibly influencing the absence-TC transition. Particularly, mutual functions among various structures of neural system are mainly modulated by the excitatory receptors and inhibitory ones such as *GABA*_*A*_ and *GABA*_*B*_ receptors performing the fast and slow time scales of inhibition effects, respectively. Presently, in order to tentatively provide the possible long and electrophysiologically observed absence-TC transition, we only considered a one layer cortical neural field model network, which is a spatially homogeneous ODE version of the original Taylor & Baier model with the addition of mutual functions of two cortical inhibitory neuronal populations. Therein, we numerically found that disinhibition may be a mechanism for the transition from tonic-clonic seizure to absence seizure, e.g. decreasing *GABA*_*A*_ inputs can induce the occurrence of spike-wave behaviour. On the other hand, Cope *et al*. (2009)^25^ experimentally considered a more realistic perspective of thalamocortical (TC) network and demonstrated that augmented rather than impaired *GABA*_*A*_ current in TC neurons of the somatosensory thalamus may be a feature of absence seizures^28^. Therefore, the fact that *GABA*_*A*_ inputs have different functions for the absence seizure in the different structure domain perplexes the mechanism for the absence-TC transition. Hence, it is still a scientific problem worthy to be further discussed. Neural system is a complex network composed of multi-layer nervous structures including the cerebral cortex and subcortical structure. In the future, more components, e.g. thalamo-cortical loop as well as the contributions via projections to the formatio reticularis also should be taken into account to explore the transitional dynamics between various epileptic seizures, which will further broaden the approach beyond the technical perspective of neural system modelling to emphasize the realistic neural network framework and its clinical correspondence.

Also, EEG recording of a patient with epilepsy evolves over time, while our results are based on the parameterization plane of coupling strengths. So the continuous-time dynamics of connectivity functions in the original model should be introduced in our future work. Whereas our results initially and qualitatively represent physiologically plausible transitions between absence and tonic-clonic seizures in the modified spatially-extended Taylor & Baier model, further research is still needed to clarify the subtleties of EEG transitions and we do hope that our paper will be motivational to that effect.

## Methods

The model considered here is a spatially-extend modified Taylor & Baier network, which is composed of several isolated Taylor & Baier neural field models on different space locations, in the form of “Mexican hat” connectivity as used in previous works^43^,^49^. The spatial extension of neural field model makes it possible to macroscopically investigate the spatiotemporal behaviors of distantly distributed neuronal populations.

### An isolated modified Taylor & Baier model

Periodic spike and slow-wave discharges are traditionally considered as the homogeneous oscillations at the macroscopic spatial scale. Therefore, previous works exclusively neglected the spatial characteristics, and only focused on the space-independent systems. To represent the periodic spike and slow-wave discharge dynamics of the cerebral cortex, Taylor & Baier^43^ extended the two-variable ODE of Amari model^49^ by adding a second inhibitory population. Hence, the resulting three-variable ODE has two competing but separate inhibitory mechanisms in this modified model. It has been confirmed that this competition can lead to robust periodic spike and slow-wave discharges dynamics^43^ when they operate on the different time-scales mediated by the inhibitory GABA_*A*_^16^,^21^ and GABA_*B*_^17^,^18^,^19^,^20^ receptors, respectively. Also, experimental result^51^ has demonstrated that there exists a basic disinhibitory circuit module in the mammalian cerebral cortex, which means that there are mutual effects among different inhibitory neurons that can not be simply ignored. Motivated by this, we incorporate the interactions between these two inhibitory populations into the modified version of Amari model^43^,^49^. The governing equations can be described as follows,


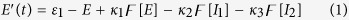



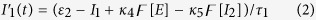



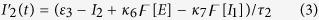


where E represents the excitatory neuronal population, I_1_ and I_2_ represent the two different types of inhibitory neuronal populations with two different fast and slow time scales, which are determined by the parameters *τ*_1_ and *τ*_2_, respectively. ε_1_,…ε_3_ are the additive constants as used in the modified version of Amari model, *κ*_1_,…*κ*_7_ are the connectivity parameters within different neuronal populations. 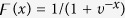 is the sigmoid transition function as used in model of Taylor & Baier^36^, where *υ* determines the steepness and *x*=E, I_1_ and I_2_.

### Spatially-extended modified Taylor & Baier model

Increasing evidence has suggested that there are significant spatial components in the generalised epileptic seizures^3^,^44^,^45^,^46^,^52^. To explore the spatial feature of neural populations, such as synchronization, the spatially-extended neural field network should be established. Following the works of Amari^49^ and Taylor & Baier^43^, by means of the form of “Mexican hat” connectivity we spatially expand the modified Taylor & Baier neural field model to much more spatial locations, thus forming the spatially-extended network. Its dynamics can be determined by the following coupled equations,


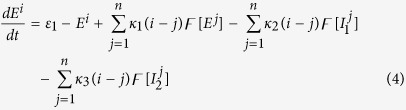



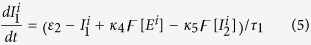



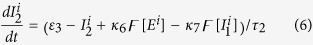


where, *i*, *j* = 1,…*n* are the number of spatial locations. Presently, we take *n* = 100 for our study. (*κ*_1_,…*κ*_7_) are the Mexican hat connectivity functions identical to the ones in Amari^49^. In our simulations, similar to Amari^49^, we use the periodic boundary conditions with one and two spatial dimensions to simulate these equations.

In addition, it is believed that cortical excitatory pyramidal neurons have sufficiently long axons which can produce significant propagation effect on the distant neuronal populations and also can be affected by these populations. However, other neural populations can only influence their adjacent areas due to the too short axons^42^. Hereby, in Eqs. (4, 5, 6), it is supposed that the excitatory populations can be coupled by the ones from all other locations, while the two inhibitory populations can only interact with the ones in the same locations.

## Additional Information

**How to cite this article**: Fan, D. *et al*. Disinhibition-induced transitions between absence and tonic-clonic epileptic seizures. *Sci. Rep*. **5**, 12618; doi: 10.1038/srep12618 (2015).
